# Getting Started in Text Mining

**DOI:** 10.1371/journal.pcbi.0040020

**Published:** 2008-01-25

**Authors:** K. Bretonnel Cohen, Lawrence Hunter

**Affiliations:** Princeton University, United States of America

## Introduction

**Text mining** is the use of automated methods for exploiting the enormous amount of knowledge available in the biomedical literature. There are at least as many motivations for doing text mining work as there are types of bioscientists. Model organism database curators have been heavy participants in the development of the field due to their need to process large numbers of publications in order to populate the many data fields for every gene in their species of interest. Bench scientists have built biomedical text mining applications to aid in the development of tools for interpreting the output of high-throughput assays and to improve searches of sequence databases (see [1] for a review). Bioscientists of every stripe have built applications to deal with the dual issues of the double-exponential growth in the scientific literature over the past few years and of the unique issues in searching PubMed/MEDLINE for genomics-related publications. A surprising phenomenon can be noted in the recent history of biomedical text mining: although several systems have been built and deployed in the past few years—Chilibot, Textpresso, and PreBIND (see Text S1 for these and most other citations), for example—the ones that are seeing high usage rates and are making productive contributions to the working lives of bioscientists have been built not by text mining specialists, but by bioscientists. We speculate on why this might be so below.

Three basic types of approaches to text mining have been prevalent in the biomedical domain. **Co-occurrence**–based methods do no more than look for concepts that occur in the same unit of text—typically a sentence, but sometimes as large as an abstract—and posit a relationship between them. (See [2] for an early co-occurrence–based system.) For example, if such a system saw that *BRCA1* and *breast cancer* occurred in the same sentence, it might assume a relationship between breast cancer and the BRCA1 gene. Some early biomedical text mining systems were co-occurrence–based, but such systems are highly error prone, and are not commonly built today. In fact, many text mining practitioners would not consider them to be text mining systems at all. Co-occurrence of concepts in a text is sometimes used as a simple baseline when evaluating more sophisticated systems; as such, they are nontrivial, since even a co-occurrence–based system must deal with variability in the ways that concepts are expressed in human-produced texts. For example, *BRCA1* could be referred to by any of its alternate symbols—*IRIS, PSCP, BRCAI, BRCC1,* or *RNF53* (or by any of their many spelling variants, which include *BRCA1, BRCA-1,* and *BRCA 1*)—or by any of the variants of its full name, viz. *breast cancer 1, early onset* (its official name per Entrez Gene and the Human Gene Nomenclature Committee), as *breast cancer susceptibility gene 1,* or as the latter's variant *breast cancer susceptibility gene-1.* Similarly, *breast cancer* could be referred to as *breast cancer, carcinoma of the breast,* or *mammary neoplasm.* These variability issues challenge more sophisticated systems, as well; we discuss ways of coping with them in Text S1.

Two more common (and more sophisticated) approaches to text mining exist: rule-based or knowledge-based approaches, and statistical or machine-learning-based approaches. The variety of types of rule-based systems is quite wide. In general, **rule-based systems** make use of some sort of knowledge. This might take the form of general knowledge about how language is structured, specific knowledge about how biologically relevant facts are stated in the biomedical literature, knowledge about the sets of things that bioscientists talk about and the kinds of relationships that they can have with one another, and the variant forms by which they might be mentioned in the literature, or any subset or combination of these. (See [3] for an early rule-based system, and [4] for a discussion of rule-based approaches to various biomedical text mining tasks.) At one end of the spectrum, a simple rule-based system might use hard-coded patterns—for example, *<gene> plays a role in <disease>* or *<disease> is associated with <gene>*—to find explicit statements about the classes of things in which the researcher is interested. At the other end of the spectrum, a rule-based system might use sophisticated linguistic and semantic analyses to recognize a wide range of possible ways of making assertions about those classes of things. It is worth noting that useful systems have been built using technologies at both ends of the spectrum, and at many points in between. In contrast, **statistical** or **machine-learning–based** systems operate by building classifiers that may operate on any level, from labelling part of speech to choosing syntactic parse trees to classifying full sentences or documents. (See [5] for an early learning-based system, and [4] for a discussion of learning-based approaches to various biomedical text mining tasks.)

Rule-based and statistical systems each have their advantages and disadvantages. For example, rule systems are often assumed (not necessarily correctly) to take a significant amount of time to develop. Statistical systems typically require large amounts of expensive-to-get labelled training data. In practice, statistical and rule-based systems can be fruitfully combined. For example, a statistical system that classifies documents as to whether or not they are relevant to the subject of genetic variation in mouse genes might use the output of a rule-based mutation recognizer as one of its feature extractors. Many systems also employ an initial statistical processing step, followed by rule-based post-processing.

A primary problem that either type of system must deal with is the issue of **ambiguity**: the existence of multiple relationships between language and meanings or categories. Ambiguity exists at every level of linguistic structure, from the part of speech of words to subtle issues in pragmatics. A common example of ambiguity in genomics text is related to gene names and symbols. Consider the string *fat*: is it an adjective, or a noun? Either part of speech is entirely plausible in biomedical texts, and PubMed returns almost 112 K hits for that single-word query (and more than 13 K even if we try to restrict the query to genomics by including the disjunction (*gene OR genetic OR genetics*). This ambiguity is relatively easy to resolve, but *fat* also turns out to be the name or symbol of a number of different genes—humans, mice, rats, *Drosophila*, zebrafish, chickens, M. mulatta, and two Lactobacilli have at least one gene whose name, official symbol, or alias is *fat.* Even if the species whose gene is being referred to can be determined, the ambiguity may still not be resolved—in humans, *fat* is the official symbol of Entrez Gene entry 2195 and an alternate symbol for Entrez Gene entry 948. The distinction is not trivial. The former is a cadhedrin, and is associated with tumor suppression and with bipolar disorder, while the latter is a thrombospondin receptor associated with atherosclerosis, platelet glycoprotein deficiency, hyperlipidemia, and insulin resistance, to name just a few phenotypes. These ambiguities are not trivial: if your analysis is wrong, you miss or erroneously extract information on relations between molecular biology and human disease.

## The First Steps: Defining Goals and Examining Data

Text mining systems can easily be as complex as any applications built in computational biology—Figure 2 at http://compbio.uchsc.edu/Hunter_lab/Cohen/Hunter_Cohen_Molecular_Cell.pdf [10] shows the levels of analysis that might be built into a representative system—and good software engineering practices can be crucial in building them successfully. An important first step is to define the desired behavior of the system. For example, consider a system that aims to extract gene/disease relations from text. Is the intended output meant for human consumption, or is it to be the input to some later automatic processing step? Is the intended input intended to be fields from a database (e.g., GeneRIFs from Entrez Gene or SUMMARY fields from Swiss-Prot), abstracts, or full-text journal articles? Each presents its own challenges and opportunities. Is the intended output lists of genes and diseases? If so, should the system make it possible to click through to the full texts from which a given gene/disease pair was extracted? Is it enough to simply output the text strings that were found in the text, or must the output be in the form of database identifiers (e.g., Entrez Gene IDs and OMIM IDs for our gene/disease example) if it is to be truly useful? Specifying these requirements early may make it possible to avoid any number of false paths in the development process.

Another important early step is potentially time-consuming, but of great importance: sitting down with a large set of potential inputs and examining them by hand. Scientists without linguistic training are often amazed at the range of linguistic possibilities for expressing even the most apparently simple biological concepts. **Four hours spent with a pile of articles and a highlighter may forestall many unpleasant surprises.**

Another important early consideration is the question of how you will evaluate your system. One basic issue arises immediately: will you be evaluating your system with an eye toward publishing a paper on it, or will you be evaluating it purely to determine its suitability for external use? For purposes of publication, the standard paradigm is to evaluate your performance on a *corpus*—a body of textual data that has been marked up with the correct answers for some task. A large number of these, suitable for a variety of different purposes, can be found at http://compbio.uchsc.edu/ccp/corpora. Recent CASP-like shared task challenges in text mining have produced a number of datasets for evaluating performance on more complex tasks, such as the assignment of Gene Ontology codes to proteins, or the detection and classification of protein–protein interactions.

For evaluation of suitability for internal use, the best paradigm might not be the publishing-oriented corpus-based one, but rather a test suite built on the principles of software engineering and structured software testing. [6] describes a methodology for doing this for a gene mention system, and discusses the advantages and disadvantages of both approaches to system evaluation. A third approach—post-hoc judging of system outputs—will often suffice for publication, but is often not practical for system development since it cannot be repeated quickly and frequently.

## Conclusions

In the introduction, we pointed out that all or most of the demonstrably useful biomedical text mining systems have been built not by text mining specialists, but by computational biologists. Why might this be? Although this has not been systematically investigated, we speculate that it is related to cultural differences between the two groups. Text mining specialists are more likely to build systems that are likely to get them published in computational linguistics conferences. Such systems are not domain-dependent, are usable for a wide variety of tasks, and, if fashionable, rely more on statistical approaches than on knowledge sources. In contrast, computational biologists do not hesitate to build systems that are extremely domain-specific, that do not attempt more than a single highly relevant task (e.g., the RLIMS-P system [7], which targets assertions about phosphorylation and nothing else), and that are not dogmatic about avoiding knowledge-based approaches. Ultimately, biologists seem to be better at one of the crucial first steps identified above: defining the goals of the system, and not hesitating to define those goals based on utility, rather than on presumed publishability in the computational linguistics literature. The key to exploiting this ability for the purpose of building a better text mining system is for the computational biologist to pay particular attention to that initial step. None of this is meant to make the claim that there is no role for computational linguists in biomedical text mining, but rather that at this time there seem to be clear roles for each. Text mining specialists continue to excel at building system components and designing datasets for evaluation; computational biologists currently appear to be much better at producing useful task definitions. Perhaps the most fruitful approaches are characterized by combined efforts that leverage the abilities of each type of scientist.

## Further Reading

Text S1 provides coverage of additional technical issues in system design and construction, and includes a number of helpful references. Additionally, text mining and natural language processing have a long history outside of the bioscience world, and have produced a sizable literature that is well worth the computational biologist's attention. [8] is an excellent starting point. [9] is the standard reference work, and is a good second step. For bioscience-specific text mining, there are a number of review papers and three useful book-length treatments. [4] takes a task-based approach to text mining, and lists a number of additional tools for most of the tasks mentioned in this short tutorial. [10] describes the state of the art, and lists a number of computational-biologist–built applications that provide good examples of high-utility systems. [11] is a collection of chapters on a wide variety of aspects of biomedical text mining. [12] focusses on document retrieval, but also contains stimulating coverage of a number of related topics in text mining. Finally, [13] provides an in-depth treatment of statistical approaches to biomedical text mining.

## Supporting Information

Text S1 Getting Started in Text Mining: Supplementary Materials (DOC 59 KB).
