# Supplementary material for: Getting Started in Text Mining
Source: PLoS Comput Biol. 2008 Jan 25;4(1):e20. doi: 10.1371/journal.pcbi.0040020 (PMC2217579; doi:10.1371/journal.pcbi.0040020)
Supplement: Text S1 — (DOC 59 KB). [file pcbi.0040020.sd001.doc]

# Getting started in text mining: supplementary materials

# K. Bretonnel Cohen and Lawrence Hunter

In this section, we expand on the material in the main body of the piece with an analysis of issues in system design and building for biomedical text mining systems.  There are *many* modules available for performing the various subtasks that we discuss in this section, and many of the ones that we omitted from mention are quite good.  The ones that we include here were selected because we have found them to be easy to use and robust with respect to operating systems, platforms, and the like.

This material supplements *Getting started in text mining,* and should be cited as *Getting started in text mining: supplementary materials,* preferably in conjunction with the DOI.

## System design: larger architectural issues

Several years ago, the only architectural option for designing a text mining system was some form of an unintegrated pipeline model: one module of the system specifies its own input and output formats, and either it is the responsibility of the next module to ensure that its input format is compatible with the output format of the preceding module, or the system developer must produce a series of scripts to convert the output formats of the various modules into the input formats of successive modules.  This approach has the advantage of presenting no overhead related to learning a global architecture, but the disadvantages of often requiring extensive work to massage formats and of being very sensitive to minor (and often difficult to detect and troubleshoot) changes in the output format of any individual module.  In the past few years, a number of architectures have been developed that facilitate the process of integrating system modules with each other.  An early option was the GATE (General Architecture for Text Engineering, Cunningham 2002) system.  It has been available since the mid 1990s, is used by many groups, and has been heavily field-tested over the years.  More recently, the UIMA (Unstructured Information Management Architecture, Ferrucci and Lally 2004, Mack et al. 2004) has seen growing popularity in the biomedical text mining community, and very active efforts to develop and distribute biomedical-domain-specific modules for it are underway at the Mayo Clinic, the Center for Computational Pharmacology at the University of Colorado School of Medicine, the National Centre for Text Mining in the United Kingdom, and the GENIA Project in the Tsujii Lab at the University of Tokyo.  

All of these integrated architectural options share a major advantage: they enforce common interfaces between system components, thereby eliminating a major (and--it cannot be emphasized enough--often difficult-to-track-down) potential source of errors.  They also share a disadvantage: committing to using any of them requires learning to use the architecture itself, and the learning curve for this can be quite long.  Also, they tend to force the use of a particular programming language; experienced users of scripting languages may decide that the disadvantages of having to switch to Java may detract considerably from the desirability of a Java-centric architecture.

LingPipe, produced by Alias-i, offers a mid-point of sorts between unintegrated pipelines and comprehensive architectures.  It is composed of a coherently organized set of modules for many biomedical (and general) language processing tasks, ranging from low-level pre-processing like sentence segmentation and part of speech tagging through complex tasks like pronoun interpretation and gene mention identification.  Much of its functionality is available through simple command-line interfaces, or as web services, obviating the need to learn a complete UIMA-like architecture just to get started.  All of the modules adhere to a common API, are serializable, and are thread-safe, so they can be (and to some extent have been) integrated into the UIMA and GATE architectures, as well (LingPipe web site).

## System design: selection of analysis modules

As we pointed out above, most or all community-accepted biomedical text mining systems have been built by computational biologists, not by text mining specialists.  The same is not true for tools that are suitable as analysis modules: these tend to be produced by language processing specialists, who may have a more nuanced understanding of the variability of human language and the computational challenges that routinely arise in dealing with it.  In this section, we recommend a number of freely available (and in some cases, Open Source) tools for carrying out these analyses.  Figure 1 (Nakov and Hearst 2005) shows a number of the levels at which an input text might need to be analyzed even prior to attempting tasks such as recognizing relations between diseases and genes.  None of these levels of analysis are trivial even in "General English" literature, and most of them present unique challenges in biomedical texts.  For example, performance on the very basic task of **sentence segmentation**--splitting a multi-sentence input into its individual sentences—varies widely, with some tools performing as poorly as 40% on this task.  High-performing systems include the rule-based Perl sentence segmenter that is packaged with the KeX gene name recognizer and the statistical Java sentence segmentation module that is distributed with LingPipe; both of them perform in the mid- to high 90s (Baumgartner et al., in progress).

**Tokenization** is the task of splitting an input into individual words (as well as other units, such as punctuation marks).  In Figure 1, this level of analysis corresponds to the *Word* level.  This task is difficult enough for General English, and presents a number of domain-specific challenges in biomedical text.  For example, hyphens can serve a number of functions in biomedical text; in some cases, they should be considered individual meaningful units, while in others, they should be considered inseparable parts of words.  In *-fever* (a common usage in clinical medical documents), the hyphen has its own semantics, indicating the absence of a fever.  In *Cl-*, it indicates a negative charge on a chlorine ion.  In *BRCA1-* it denotes a non-functional gene, while in *BRCA1-IRIS* (PMID 15448696) it joins two synonymous forms of the gene name, and in *BRCA1-mediated* its use is syntactic, not semantic, and indicates a particular relationship between the word *BRCA1* and the word *mediated,* in which *BRCA1* is the "agent" of the action of mediation.  In *BRCA1-CtIP,* the hyphen has yet another role, indicating that the BRCA1 protein forms a complex with the CtIP substrate of ATM protein kinase.  Thus, we see at least six separate uses of the hyphen in biomedical text, with a variety of syntactic, semantic, and mixed syntactic/semantic functions, some of which should be "tokenized" into separate meaningful units, and some of which should not.  Texts containing chemical names contain an additional set of challenges of their own; ChemTok (Corbett et al. 2007) is specialized for this type of input.

**Abbreviations** and the related phenomenon of gene symbol definitions are ubiquitous in biomedical texts.  Two systems for handling this issue are quite popular.  Many system developers implement Schwartz and Hearst's algorithm for locating abbreviation/symbol definitions within input texts (Schwartz and Hearst 2003); this approach has the advantage of being easily implementable in Java, of returning a single output with a high likelihood of being correct, and of requiring no connections with external applications.  Another popular approach involves using Chang et al.'s remote abbreviation server (Chang et al. 2002).  This approach has the advantage of requiring no re-implementation of the algorithm, and of allowing for use via any language with a facility for remote procedure calls.

**Gene mention** recognition is a required component for almost any genomically relevant text mining application.  Because of both its central role in most applications and its surprising difficulty, it has been a major focus of biomedical text mining research.  Although *many* systems have been built for carrying out this important task, most of them are not publicly available.  One popular exception is the ABNER system (Settles 2005).  Its advantages include robustness (it is distributed as a .jar file and works well on multiple platforms) and a variety of input and output format options.  In addition to gene/protein names, it also locates cell types and cell lines.  Another popular choice is LingPipe's gene-mention-finding module.

For the recognition of a wider range of biomedically relevant categories--diseases, drugs, chemicals, treatment modalities, and the like--a popular choice is National Library of Medicine's MetaMap tool (Aronson 2001).  MetaMap is available as a Java API or via a server.  It recognizes variant forms of domain-relevant concepts; for example, it would recognize that the several ways of referring to breast cancer mentioned earlier are all different forms of that concept.

A number of possibilities exist for more purely linguistic analyses.  The GENIA Lab at the University of Tokyo makes available a set of tools for labelling the part of speech of individual words, and for performing syntactic analysis of sentences; all of these are customized for dealing with biomedical text.


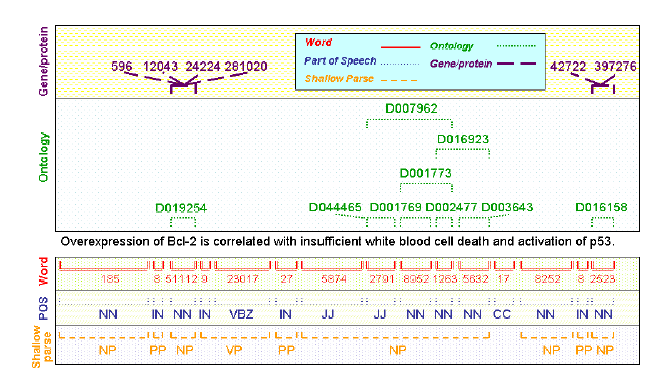


**Figure 1: Levels of analysis.  From Nakov and Hearst (2005).**

## References for supplementary materials

Aronson, Alan R. (2001)  Effective mapping of biomedical text to the UMLS Metathesaurus: the MetaMap program.  *Proceedings of the American Medical Informatics Association,* pp. 17-21.  American Medical Informatics Association.

Chang, Jeffrey T.; Heinrich Schuetze; and Russ B. Altman (2002)  Creating an online dictionary of abbreviations from MEDLINE.  *Journal of the American Medical Informatics Association* 9(6):612-620.
 
Corbett, Peter; Colin Batchelor; and Simone Teufel (2007)  Annotation of chemical named entities.  *BioNLP 2007: Biological, translational, and clinical language processing,* pp. 57-64.  Association for Computational Linguistics.

Cunningham, Hamish (2002) GATE, a general architecture for text engineering. *Computers and the Humanities* 36(2):223-254.

Ferrucci, David; and Adam Lally (2004) UIMA: An architectural approach to unstructured information processing in the corporate research environment. *Natural Language Engineering* 10(3/4):327-348.

LingPipe web site (undated) http://www.alias-i.com/lingpipe/

Mack, R.; S. Mukherjea; A. Soffer; N. Uramoto; E. Brown; A. Coden; J. Cooper; A. Inokuchi; B. Iyer; Y. Mass; et al. (2004) Text analytics for life science using the unstructured information management architecture. *IBM Systems Journal* 43(3):490-515.

Nakov, Preslav; Ariel Schwartz; B. Wolf; and Marti A. Hearst (2005)  Supporting annotation layers for natural language processing.  *Proceedings of the ACL interactive poster and demonstration sessions.* Association for Computational Linguistics.

Schwartz, Ariel; and Marti A. Hearst (2003)  A simple algorithm for identifying abbreviations definitions in biomedical text.  *Pacific Symposium on Biocomputing* 8:451-462.

Settles, Burr (2005) ABNER: an open source tool for automatically tagging genes, proteins, and other entity names in text.  *Bioinformatics* 21(14):3191-3192.
